# Identification of Hub Genes for Psoriasis and Cancer by Bioinformatic Analysis

**DOI:** 10.1155/2024/5058607

**Published:** 2024-07-16

**Authors:** Yao Yu, Shaoze Ma, Jinzhe Zhou

**Affiliations:** ^1^ Department of Dermatology Shanghai Putuo District Liqun Hospital, Shanghai 200333, China; ^2^ Department of Urology Surgery Baoshan Branch of Shanghai Hospital Affiliated to Shanghai University of Traditional Chinese Medicine, Shanghai 201999, China; ^3^ Department of General Surgery Tongji Hospital Tongji University School of Medicine, Shanghai 200065, China

**Keywords:** bioinformatic analysis, cancer, hub genes, psoriasis

## Abstract

Psoriasis increases the risk of developing various cancers, including colon cancer. The pathogenesis of the co-occurrence of psoriasis and cancer is not yet clear. This study is aimed at analyzing the pathogenesis of psoriasis combined with cancer by bioinformatic analysis. Skin tissue data from psoriasis (GSE117239) and intestinal tissue data from colon cancer (GSE44076) were downloaded from the GEO database. One thousand two hundred ninety-six common differentially expressed genes and 688 common shared genes for psoriasis and colon cancer were determined, respectively, using the limma R package and weighted gene coexpression network analysis (WGCNA) methods. The results of the GO and KEGG enrichment analyses were mainly related to the biological processes of the cell cycle. Thirteen hub genes were selected, including AURKA, DLGAP5, NCAPG, CCNB1, NDC80, BUB1B, TTK, CCNB2, AURKB, TOP2A, ASPM, BUB1, and KIF20A. These hub genes have high diagnostic value, and most of them are positively correlated with activated CD4 T cells. Three hub transcription factors (TFs) were also predicted: E2F1, E2F3, and BRCA1. These hub genes and hub TFs are highly expressed in various cancers. Furthermore, 251 drugs were predicted, and some of them overlap with existing therapeutic drugs for psoriasis or colon cancer. This study revealed some genetic mechanisms of psoriasis and cancer by bioinformatic analysis. These hub genes, hub TFs, and predicted drugs may provide new perspectives for further research on the mechanism and treatment.

## 1. Introduction

Psoriasis is a prevalent inflammatory and immune-mediated disease identified by the presence of erythematous scaly lesions. Extensive studies have indicated that people with psoriasis are confront with an increased risk of developing various cancers, such as lung cancer, bladder cancer (BLCA), hepatobiliary cancer, colorectal cancer, rectal cancer, squamous cell carcinoma, and lymphoma [1–4]. Not only that, patients with psoriasis also have an increased risk of cancer-related mortality [4]. Further research has suggested that increased susceptibility to cancer in psoriasis patients is considered to be affected by factors, such as impaired immune surveillance, immunomodulatory therapy, chronic inflammation, obesity, and other commonly shared risk factors [3, 5]. Abnormal immunoinflammatory microenvironment and cell proliferation form a vicious cycle that plays a role in psoriasis and cancer, promoting keratinocyte proliferation in psoriasis and tumor cell proliferation in cancer [6–8]. In the past decades, studies on the mechanism of the two diseases have mainly focused on immune and inflammatory abnormalities. The underlying genetic mechanism remains unclear.

Furthermore, there is limited research on the treatment of patients with both psoriasis and cancer simultaneously. Case reports and small-sample retrospective analyses suggest that biologics and combination chemotherapy may effectively address the treatment of both conditions, but the evidence for these studies is relatively low [9, 10]. There is controversy regarding whether treatment for psoriasis increases the risk of cancer. Some studies suggest that certain medications used to treat psoriasis may increase the risk of cancer [11]. However, large retrospective analyses suggest that this increased risk of cancer in psoriasis is independent of systemic treatment for psoriasis [12]. Therefore, this study is aimed at further predicting therapeutic drugs while investigating the shared genetic mechanisms of both conditions.

Colon cancer is a common gastrointestinal tumor, and many studies have found that the incidence of colon cancer is higher among patients with psoriasis [2, 3]. A retrospective analysis involving 32,910 patients studied the risk of cancer in psoriasis patients. The results showed that, after adjustment for age, psoriasis was associated with an increased risk of lung cancer and colon cancer. However, after further adjustment for smoking, body mass index, education level, physical activity, and hormone therapy, only the association with colon cancer remained statistically significant, particularly stronger with severe psoriasis [13]. Additionally, a meta-analysis also indicates an increased risk of colon cancer in patients with severe psoriasis [4]. So, it can be an effective model to reveal the interrelation linking psoriasis and cancer. Not only that, but the shared genetic mechanisms between psoriasis and colon cancer are not yet clear, and related studies are scarce. We approach the study with psoriasis and colon cancer as focal points. Detailed investigation of the common genetic mechanism of psoriasis and cancer using the bioinformatics method explored the genetic and transcriptional factors of psoriasis and its associated cancers, as well as predict potential therapeutic drugs.

## 2. Materials and Methods

### 2.1. Selection of Data Sets

The psoriasis and colon cancer data sets were obtained from the GEO database (https://www.ncbi.nlm.nih.gov). The selection process involved first sorting the GEO data by sample size in descending order. Then, preliminary analysis was performed using the GEO2R tool provided by the GEO database, and the resulting PCA plot was examined. Data sets in which the case group and the healthy control group were clearly separated in the PCA plot, without any confounding, were chosen as the final research data sets. The psoriasis and healthy control skin tissue data set GSE117239 include 83 psoriasis samples and 84 control samples, and the colon cancer and healthy control intestinal tissue data set GSE44076 include 98 colon cancer samples and 50 control samples.

### 2.2. Differential Gene and Shared Gene Analysis

Differential gene and shared gene analyses for the two data sets were conducted using the limma R package and the weighted gene coexpression network analysis (WGCNA) method, respectively.

Differential gene analysis for both data sets was conducted using the “GEOquery” and “limma” packages in the R4.2.22 version software. The threshold was set to adj. *p*.Val < 0.05 and an absolute value of logFC > 0.5. After data standardization, for psoriasis and colon cancer, clustering and heat map visualization were performed, and abnormal samples were removed. In the psoriasis group, five samples (GSM289572, GSM289565, GSM289597, GSM289591, and GSM289604) were excluded, leaving 78 samples in the psoriasis group and 84 samples in the control group. Similarly, a sample (GSM1077800) was removed from the colon cancer group, leaving 97 samples in the colon cancer group and 50 samples in the control group. The differentially expressed genes obtained from the analysis were categorized into low-expression and high-expression genes, and the intersection genes were taken using a Venn diagram as the common differentially expressed genes for the limma R package analysis of psoriasis and colon cancer.

Using the WGCNA R package in R4.2.22, the modules were identified in the combined matrices of the two data sets. WGCNA is a bioinformatic analysis method used to describe gene association patterns among different samples. It can cluster genes with similar expression patterns and analyze the association between modules and specific traits or phenotypes [14]. Outlier samples were excluded, and the soft threshold values for the psoriasis data set were set to *β* = 9 (signed *R*^2^ > 0.9), with a minimum module size of 30. The soft threshold value for the colon cancer data set was set to *β* = 14 (signed *R*^2^ > 0.9), with a minimum module size of 30. Modules with *p* < 0.05 were considered as containing shared genes. The shared genes in psoriasis and colon cancer were divided into low- and high-expression genes based on different modules, and the overlapping genes were selected using a Venn diagram as the common shared genes for the WGCNA.

The differential genes and shared genes obtained through the limma R package and WGCNA method were intersected using a Venn diagram. These intersecting genes are considered shared differential genes.

### 2.3. GO and KEGG Enrichment Analyses

The differential genes obtained from the limma R package underwent GO enrichment analysis (*p*valueCutoff = 0.05, *q*valueCutoff = 0.05) and KEGG enrichment analysis (*p*valueCutoff = 0.05, *q*valueCutoff = 0.05) using the “DOSE” and “clusterProfiler” packages in the R version 4.2.2 software.

### 2.4. Construction of PPI Network and Selection of Hub Genes

The PPI network was constructed using the STRING website (https://cn.string-db.org/cgi/input?sessionId=bP20FGDFn7qe%26input_page_show_search=on) with a high confidence score > 0.7. The weights of the differential genes and shared genes obtained from the limma R package and the WGCNA method were calculated using the MCC algorithm in the Cytoscape cytoHubba plugin. In the coexpression network, the MCC algorithm is considered the most effective method for identifying key nodes [15]. The top-ranked gene for each method was obtained, and the intersection was taken using a Venn diagram to determine the hub genes that are commonly associated with both psoriasis and colon cancer. The GeneMANIA website (http://genemania.org/) was used for further analysis of the hub genes.

### 2.5. Validation of Hub Genes

For validation purposes, GSE14905 (33 samples in the psoriasis group and 21 samples in the healthy control group) for psoriasis and GSE10972 (24 samples in the colon cancer group and 24 samples in the healthy control group) for colon cancer were acquired from the GEO database (https://www.ncbi.nlm.nih.gov), respectively. The “GEOquery,” “limma,” and “ggplot2” packages in R version 4.2.22 were employed to analyze whether the hub genes were differentially expressed between the psoriasis group and the healthy control group, as well as between the colon cancer group and the healthy control group. The threshold set as adj. *p*.Val < 0.05 and the absolute value of logFC > 0.5.

### 2.6. Diagnostic Value of the Hub Genes

The diagnostic value of the hub genes in the GSE117239 and GSE44076 data sets was determined by calculating the area under the curve (AUC) of the receiver operating characteristic (ROC) curve. This analysis was performed using the “pROC” package in R version 4.2.2.

### 2.7. Hub Genes and Immune Infiltration

The correlation between hub genes in psoriasis and colon cancer and the abundance of 28 immune cells were analyzed by using the “GSVA” R package to perform ssGSEA. The correlation analysis was conducted using the Spearman rank correlation test (*p* < 0.05).

### 2.8. Expression Profiles of Hub Genes in Multiple Cancers

The expression of hub genes in various malignant tumors and paired normal tissues was obtained from the GEPIA2 database (http://gepia2.cancer-pku).

### 2.9. Predicting Transcription Factors (TFs) and Verifying Their Expression in Psoriasis and Cancer

Using hub genes, TFs were predicted through the TRRUST website (https://www.grnpedia.org/trrust/Network_search_form.php), and the differential expression of predicted TF genes in the GSE117239 and GSE44076 data sets was validated. The validated TFs were considered as the hub TFs. Furthermore, the expression of hub TF genes in various cancers and paired normal tissues was acquired from the GEPIA2 database (http://gepia2.cancer-pku).

### 2.10. Drug Prediction

The existing therapeutic drugs for psoriasis and colon cancer were investigated through the MalaCards website (https://www.malacards.org/). Drugs based on hub genes and hub TFs were predicted using the DGIdb website (https://www.dgidb.org/). The drug-gene interaction database (DGIdb, http://www.dgidb.org) consolidates, organizes, and presents drug-gene interactions and gene druggability information from papers, databases, and web resources. Comparison of the existing therapeutic drugs with predicted drugs was performed using a Venn diagram.

## 3. Results

### 3.1. Differential Genes and Shared Genes in Psoriasis and Colon Cancer and Their Coexpressed Differentially Shared Genes

**Figure 1:**
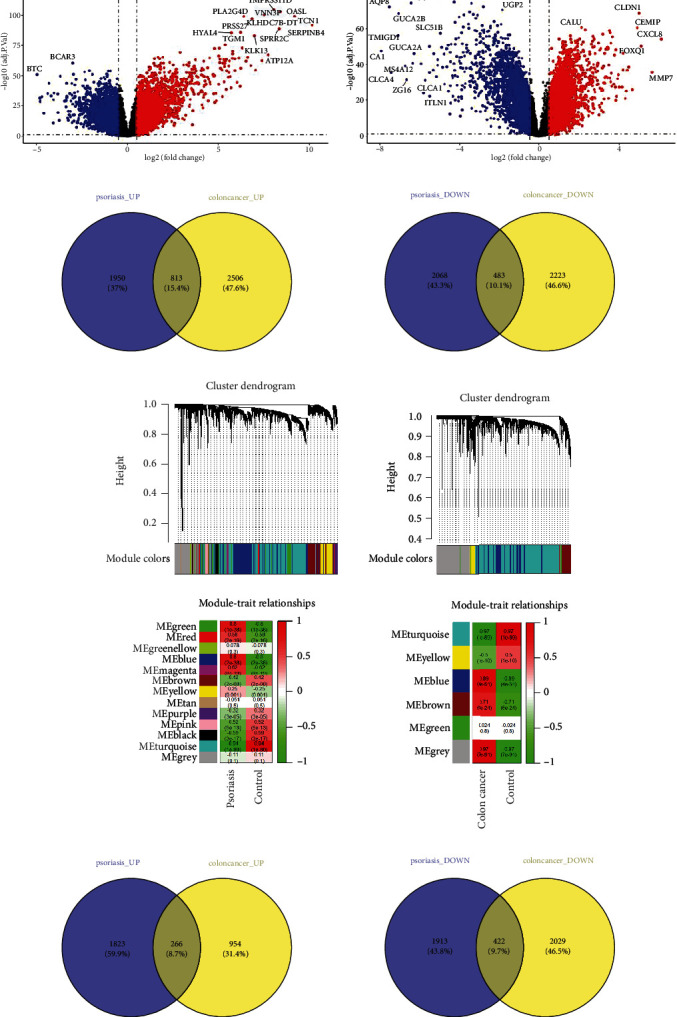
Differential and common genes in psoriasis and colon cancer data sets. (a) Volcano plots illustrate the differential gene expressions in psoriasis (left) and colon cancer (right); red indicates upregulated genes, and blue indicates downregulated genes. (b) Venn diagrams show commonly differentially expressed genes in both psoriasis and colon cancer by the limma R package. (c) WGCNA identifies and analyzes shared genes in psoriasis and colon cancer data sets. The upper cluster dendrogram shows the hierarchical clustering tree of genes, and the lower one shows the gene modules. Correlations between MEs and groups indicating the associations of the module trait. The row represents a ME, and the column represents the group. (d) Venn diagrams show that 266 shared genes are commonly upregulated and 422 are commonly downregulated between psoriasis and colon cancer, as identified by WGCNA.

The differential genes were analyzed using the limma R package, and the results showed that 5314 differential genes were identified in psoriasis, with 2763 upregulated genes and 2551 downregulated genes. In the case of colon cancer, there were 6025 differential genes, including 3319 upregulated genes and 2706 downregulated genes recognized (Figure 1(a)). Among these, there were 813 differential genes commonly upregulated and 483 differential genes commonly downregulated, with a total of 1296 differential genes common in both psoriasis and colon cancer (Figure 1(b)).

Furthermore, the WGCNA method was employed to further analyze shared genes and is shown in Figure 1(c). It revealed that 4424 shared genes in psoriasis were identified, including 2089 upregulated genes and 2335 downregulated genes. In the case of colon cancer, there were 3671 shared genes, including 1220 upregulated genes and 2451 downregulated genes. Among these, there were 266 shared genes commonly upregulated and 422 shared genes commonly downregulated, with a total of 688 shared genes common in both psoriasis and colon cancer (Figure 1(d)).

### 3.2. GO and KEGG Enrichment Analyses and PPI Construction

**Figure 2:**
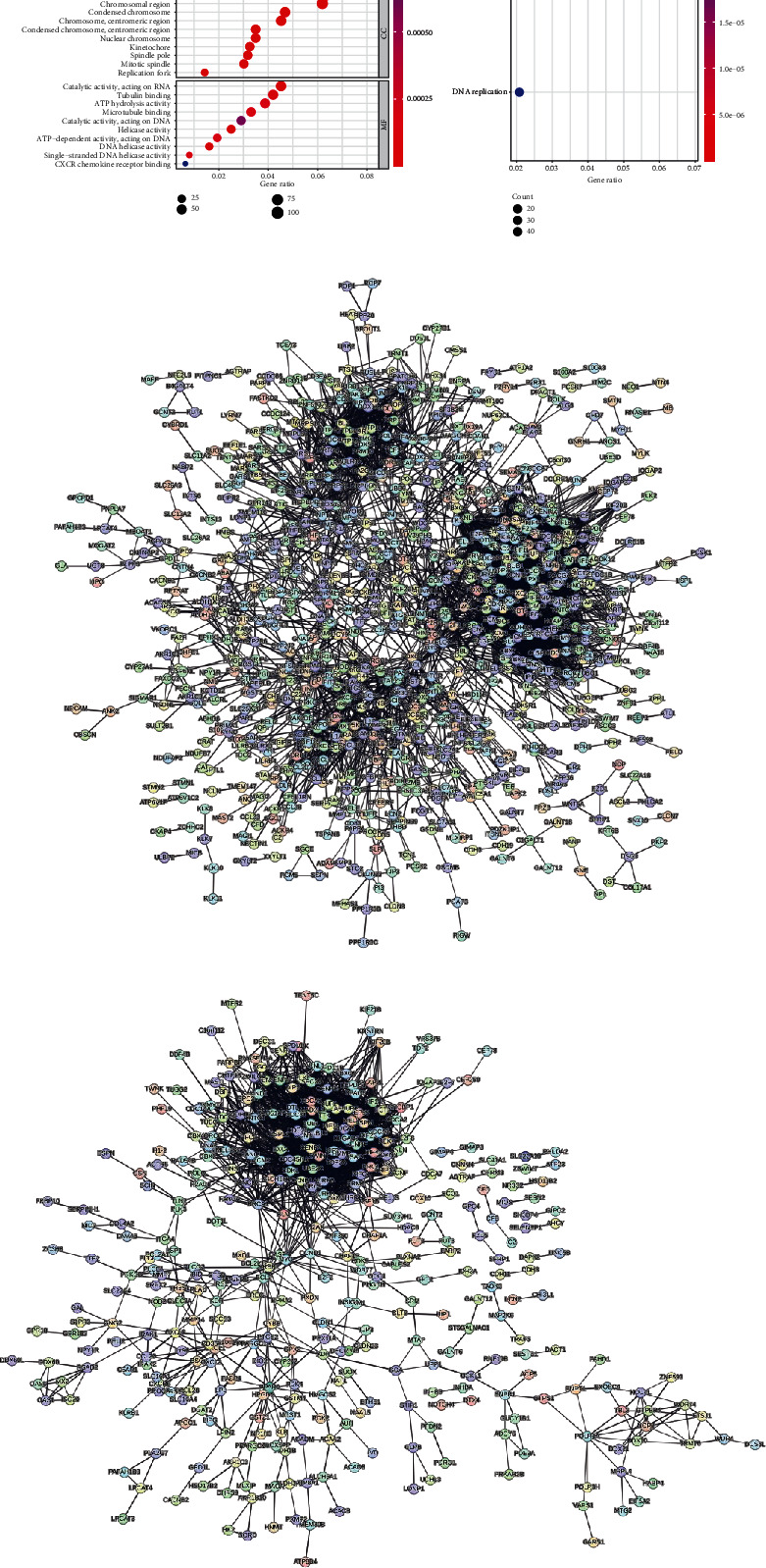
GO and KEGG enrichment analyses and PPI construction. (a) GO enrichment analysis for differentially expressed genes obtained using the limma R package method. The size of the circles represents the number of genes involved, and the horizontal axis represents the frequency of the genes. The results of the KEGG enrichment analysis are also shown, where the size of the circles represents the number of genes involved and the horizontal axis represents the frequency of the genes. (b) PPI network constructed using differentially expressed genes obtained using the limma R package. (c) PPI network constructed using shared genes obtained using the WGCNA methods.

The differential genes obtained using the limma R package were subjected to GO enrichment analysis, with results sorted from small to large based on the *p*.adjust value and GeneRatio (Figure 2(a)): In the BP category, the top 3 pathways with lower p.adjust values and more enriched genes were nuclear division, mitotic nuclear division, and DNA replication. In the CC category, the top 3 terms were chromosomal region, spindle, and chromosome, centromeric region. In the MF category, the top 3 terms were catalytic activity, acting on RNA, helicase activity, and DNA helicase activity. The KEGG enrichment analysis focuses on the cell cycle and DNA replication pathways (Figure 2(a)).

GO and KEGG enrichment analyses primarily focused on the biological processes of the cell cycle.

A PPI network was constructed using the STRING website (Figures 2(b) and 2(c)). Using the cytoHubba plugin in Cytoscape, 16 genes from differentially expressed genes obtained using the limma R package method and 18 genes from shared genes obtained with the WGCNA method achieved the highest score. A Venn diagram was used to obtain the intersection, resulting in 13 hub genes, which are AURKA, DLGAP5, NCAPG, CCNB1, NDC80, BUB1B, TTK, CCNB2, AURKB, TOP2A, ASPM, BUB1, and KIF20A (Table 1). GeneMANIA analysis revealed a high degree of coexpression among these hub genes (63.72%), while physical interactions and predicted interactions accounted for 13.96% and 12.82%, respectively.

### 3.3. Analysis of Hub Genes in Psoriasis and Cancer

**Figure 3:**
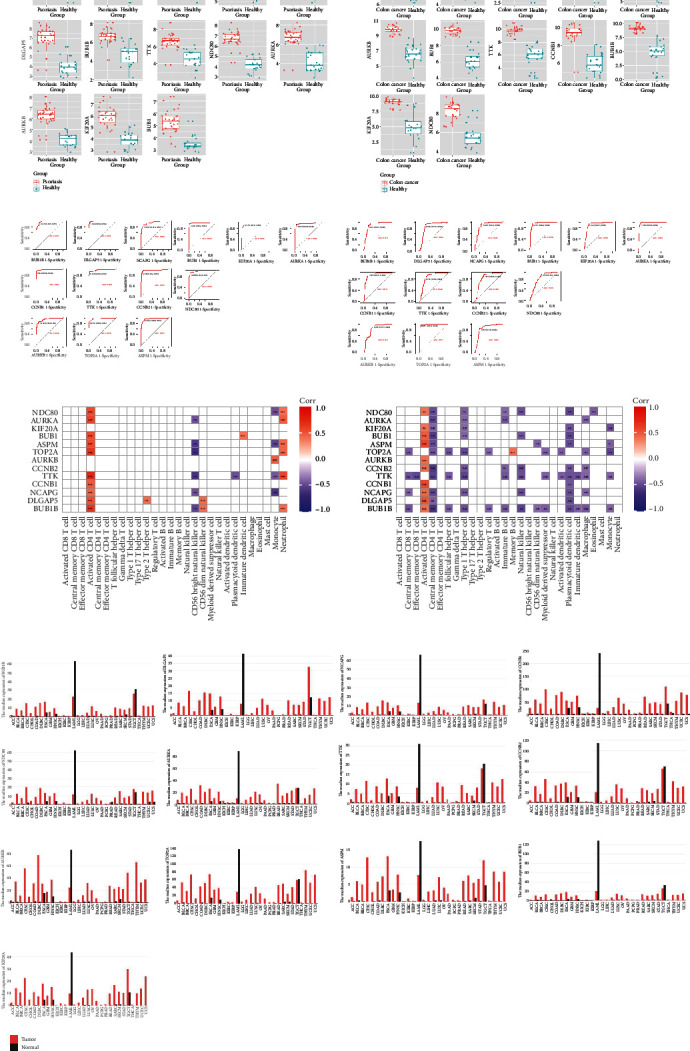
Identification of hub genes. (a) Validation of hub genes. Expression levels of hub genes were significantly upregulated in skin tissue from psoriasis (GSE14905) and colon tissue from colon cancer (GSE10972) compared to normal healthy control groups. The DLGAP5 gene was not present in the GSE10972 data set. (b) Diagnostic value of hub genes. ROC curve of the 13 hub genes in psoriasis (left) and ROC curve of the 13 hub genes in colon cancer (right). (c) Immune infiltration of hub genes. Correlation plot showing the correlation between hub genes and the abundance of 28 immune cells in psoriasis and colon cancer. (d) Hub genes in other cancers. The 13 bar chart from the GEPIA2 database shows the expression profiles of the hub genes in various types of tumor samples and paired normal tissues. The height of the bars represents the median expression in a particular tumor type or normal tissue, and the horizontal axis represents the name of the tumor.

Validation experiments were conducted to assess the expression levels of the 13 hub genes in psoriasis skin tissue (GSE14905) and colon tissue from patients with colon cancer (GSE10972), as compared to normal healthy control groups. The results showed significant upregulation (adj. *p*.Val < 0.05) of these hub genes, except for the DLGAP5 gene which was not present in the colon cancer data set (Figure 3(a)).

Furthermore, the 13 hub genes showed high sensitivity and specificity in distinguishing psoriasis (Figure 3(b), left). In colon cancer, all hub genes showed high sensitivity and specificity, except NDC80, which was not present in the data set (Figure 3(b), right).

Correlation analysis showed associations between the expression of hub genes in psoriasis and colon cancer and certain immune cells. Most of the hub genes showed a positive correlation with activated CD4 T cells (Figure 3(c)).

Bar graphs acquired from the GEPIA2 database revealed abnormal expression patterns of hub genes in various tumor types (Figure 3(d)). Notably, these hub genes exhibited particularly high expression in tumors related to psoriasis, such as lung adenocarcinoma (LUAD), lung squamous cell carcinoma (LUSC), BLCA, liver cancer (LIHC), gallbladder cancer (CHOL), colon cancer (COAD), rectal cancer (READ), head and neck squamous cell carcinoma (HNSC), lymphoma (DLBC), and esophageal carcinoma (ESCA).

### 3.4. Hub TFs and Drug Prediction

**Figure 4:**
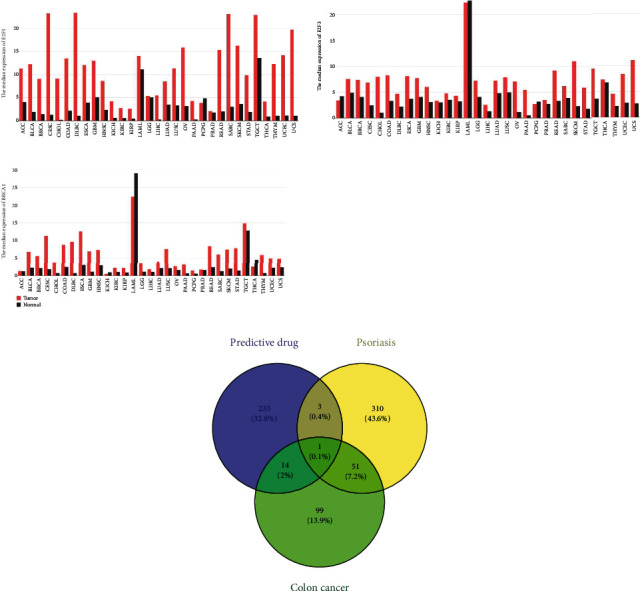
Hub TFs and drug prediction. (a) Bar charts from the GEPIA2 database showing the expression profiles of the hub TFs in various types of tumor samples and matched normal tissues. The height of the bars represents the median expression in a particular tumor type or normal tissue, and the horizontal axis indicates the tumor name. (b) Venn diagram showing the predicted drugs and existing therapeutic drugs for psoriasis and colon cancer.

The TRRUST website predicted six hub TFs, including E2F4, E2F1, E2F3, YBX1, BRCA1, and TP53 (Table 2). Among them, three TF genes, E2F1, E2F3, and BRCA1, were validated as differentially expressed in databases of psoriasis and colorectal cancer, confirming their designation as hub TFs. Analysis from the GEPIA2 database revealed aberrant expression of these three hub TF genes in various tumor samples (Figure 4(a)).

The MalaCards website indicated that there are 365 existing therapeutic drugs for psoriasis and 165 existing therapeutic drugs for colon cancer. Additionally, there were 251 drugs predicted through the DGIdb website. Notably, paclitaxel was identified as a drug that demonstrates a common effect on both psoriasis and colon cancer. Among the predicted drugs, methotrexate, riboflavin, and vitamin B2 have been used for the treatment of psoriasis, but not for the treatment of colon cancer. On the other hand, sorafenib, cisplatin, fluorouracil, erlotinib, dasatinib, doxorubicin, etoposide, etoposide phosphate, hydroquinone, irinotecan, resveratrol, vinorelbine, oxaliplatin, and gemcitabine have been used for colon cancer treatment, but not for psoriasis treatment (Figure 4(b)).

## 4. Discussion

In this study, we employed two methods, namely, limma R package and WGCNA, to analyze the differentially expressed genes and shared genes in psoriasis, colon cancer, and normal populations. The differentially expressed genes were then subjected to GO and KEGG enrichment analyses using R language. The enrichment analysis revealed that these genes were primarily involved in biological processes related to the cell cycle, such as cell cycle, DNA replication, and DNA helicase activity. These findings are line with the clinical manifestations of psoriasis (epidermal cell proliferation) and colon cancer (tumor cell proliferation).

Furthermore, 13 hub genes were identified with this analysis, namely, AURKA, DLGAP5, NCAPG, CCNB1, NDC80, BUB1B, TTK, CCNB2, AURKB, TOP2A, ASPM, BUB1, and KIF20A. The analysis of the ROC curve demonstrated that these hub genes exhibited high sensitivity and specificity in the diagnosis of psoriasis. With all hub genes, except NDC80, they also show high sensitivity and specificity in colon cancer. The correlation analysis also revealed associations between the expression of these central genes and certain immune cells, which predominantly show positive correlations with activated CD4 T cells.

Additionally, an increased risk of various cancers was observed in patients with psoriasis, including lung cancer LUAD\LUSC, BLCA, liver cancer (LIHC), gallbladder cancer (CHOL), colon cancer (COAD), rectal cancer (READ), HNSC, lymphoma (DLBC), and ESCA. The GEPIA2 database confirmed the high expression of hub genes in these cancers.

It is noteworthy that these hub genes all function in the biological processes of the cell cycle, which are closely associated with cancers; however, their relationships with psoriasis have barely been investigated, such as AURKA and AURKB belong to the serine/threonine kinase family and are involved in regulating the mitotic process. The activation of AURKA and AURKB has been shown to play an important role in multiple cancers, and their overexpression is generally associated with tumor cell invasion and metastasis. Currently, some small-molecule drugs targeting AURKA and AURKB have been discovered for the treatment of cancer [16, 17]. We found that all of these hub genes all act on the biological processes of the cell cycle, which is closely related to cancer, but there is less research on their relationship with psoriasis. AURKA and AURKB belong to the serine/threonine kinase family and participate in the regulation of the mitosis process. The activation of AURKA and AURKB has been shown to play an important role in multiple cancers, and their overexpression is generally associated with tumor cell invasion and metastasis. Currently, some small-molecule drugs targeting AURKA and AURKB have been discovered for cancer treatment [16, 17].

One study found that AURKA is highly expressed in psoriasis tissues and can promote the occurrence of psoriasis-related inflammation by blocking the autophagy-mediated AIM2 inflammasome [18]. There is less research on the role of AURKB in psoriasis. KIF20A is a member of the kinesin family, which transports chromosomes during mitosis and plays a critical role in cell division. In recent years, studies have confirmed that KIF20A is highly expressed in cancer [19]. High expression of KIF20A promotes tumor proliferation, migration, and invasion and is associated with poor overall survival. In psoriasis, inhibition of miR-214-3p can increase the expression of genes such as KIF20A, leading to excessive proliferation and increased apoptosis of in vitro keratinocytes [20]. BUB1 is a mitotic checkpoint serine/threonine kinase that promotes cell proliferation in various cancers [21]. A pan-cancer investigation of the BUB1B gene revealed its significant role in cancer, which is related to immunology, cancer stem cells, and genetic alterations in multiple cancer types [22]. CCNB1 and CCNB2 are members of the cell cycle protein family and play important roles in signal transduction during mitosis. ASPM is involved in the regulation of the mitotic spindle during cell replication. DLGAP is a kinetochore fiber-binding protein that functions as an oncoprotein in many cancers. Overexpression of CCNB1, CCNB2, DLGAP5, and ASPM is associated with tumor progression and poor prognosis [23–27]. NCAPG is a chromosome condensation protein associated with mitosis and meiosis, responsible for the condensation and stability of chromosomes during these processes. Studies have shown that NCAPG has a highly regulatory role in multiple cancers, and its related molecular mechanisms affect tumor cell proliferation, invasion, metastasis, and apoptosis, including liver cancer, prostate cancer, breast cancer, gastric cancer, glioma, LUAD, colorectal cancer, ovarian cancer, and endometrial cancer, among others [28]. NDC80 is a subunit of the NDC80 complex and forms and stabilizes the attachment of microtubules to kinetochores during chromosome segregation in mitosis [29]. TTK is a serine/threonine kinase that plays an important role in the signaling of the spindle assembly checkpoint. TOP2A is a subtype of type II topoisomerase (TOP2) and is a conserved regulator of chromatin topology. It catalyzes reversible DNA double-strand breaks and is involved in maintaining genome integrity in various dynamic processes such as transcription, replication, and cell division [30]. All of these genes are associated with the occurrence and development of multiple cancers [30–34]. However, there is little research on their association with psoriasis, and further studies are needed to determine their specific roles and mechanisms in psoriasis.

Furthermore, through verification on the TRRUST website and the GEO database, three hub TFs were acquired, namely, E2F1, E2F3, and BRCA1. The GEPIA2 database confirmed that the genes of these three hub TFs are also abnormally expressed in various types of tumor samples. E2F1 and E2F3 are members of the E2Fs TF family, which can participate in the cell cycle and DNA synthesis by regulating the expression of genes dependent on the cell cycle–dependent genes [35]. They affect the invasion and prognosis of various cancers [36, 37]. E2F1 and E2F3 binds to DNA with dimerization partner (DP) proteins through an E2 recognition site. E2F1 and E2F3 regulate the expression of several genes associated with cell proliferation, apoptosis, and differentiation, primarily in a cell cycle–dependent manner [38]. The activity of E2F1 and E2F3 is typically regulated through the retinoblastoma protein (pRb), cyclins, p53, and the ubiquitin-proteasome pathway [38]. E2F1 can regulate the activity of p53 and its homolog TAp73 and promote cell apoptosis by activating excessive apoptotic pathways [39]. E2F1 has been shown to be involved in cell proliferation, differentiation, and apoptosis in various cancers, as well as tumor metastasis and chemotherapy resistance [40]. The E2F3 TF drives the cell cycle from G1 to S phase, and alterations in E2F3 function are also associated with poor prognosis in various cancers [41]. In colorectal cancer, E2F3 acts as a promoter-regulated factor, exacerbating tumor occurrence during colorectal cancer progression through the STAT3 pathway [42]. Furthermore, high levels of E2F3 expression are associated with reduced overall survival and disease-free survival rates in colorectal cancer patients, showing significant correlation with cancer staging [43].

The BRCA1 TF participates in various biological pathways such as DNA damage response, transcription regulation, cell growth, and apoptosis [44]. Its encoding gene is a tumor suppressor gene, and its mutations are associated with the development of breast cancer, ovarian cancer, and other malignant tumors at a young age. Protein kinases involved in DNA damage checkpoint control, such as ATM, ATR, and hCds1/Chk2, have been shown to phosphorylate and activate BRCA1 upon DNA damage. The phosphorylation status of BRCA1 is closely associated with its functions in cell cycle regulation and DNA repair [45]. The recruitment of BRCA1 to DNA break sites relies on RAP80 and RNF8/RNF168 E3 ubiquitin ligases [46]. Additionally, BRCA1 can regulate the transcription of various genes by interacting with RNA polymerase II holoenzyme complex, exerting activating or inhibitory effects. For instance, as an activator, BRCA1 induces the transcriptional activity of genes involved in nucleotide excision repair, such as XPC, DDB2, and GADD45. There are also reports suggesting that BRCA1 may participate in the silencing of heterochromatic transcription through its E3 ubiquitin ligase activity [47]. The roles of these three hub TFs in psoriasis still require further research.

In the study, 251 drugs were predicted using the DGIdb website. Among them, methotrexate, riboflavin, and vitamin B2 have been used for the treatment of psoriasis, but not for the treatment of colon cancer. Some of these drugs show potential use for colon cancer treatment. Methotrexate primarily regulates inflammation and immunity by acting on the folate pathway, adenosine, prostaglandins, leukotrienes, and cytokines [48]. High-dose methotrexate is used to treat a range of cancers, such as lymphoma, choriocarcinoma, and breast cancer. It is safe for most patients, while it can cause severe nephrotoxicity [48, 49]. Numerous studies have shown that riboflavin is associated with cancer, with riboflavin deficiency increasing the risk of developing cancer [50]. On the other hand, high-dose riboflavin supplementation has been shown to reverse the effects of hepatocarcinogens in rodents, although the specific mechanisms remain unclear [51]. Vitamin B2 is a cofactor that facilitates one-carbon metabolism and maintains mucosal integrity. The vitamin B complex has the ability to modulate immune and inflammatory responses. Studies have found that vitamin B2 can exert antitumor effects on monocytic precursor lymphoma cells by altering proliferation, migration, apoptosis, cytokine levels, and the expression of programmed death-ligand 1 (PD-L1) [52]. Other studies have found that vitamin B2 is a promising sensitizer, capable of enhancing the efficacy of vitamin C–based cancer chemoprevention and chemotherapy by inhibiting the phosphorylation of Akt and Bad [53]. A meta-analysis indicates that vitamin B2 intake is inversely correlated with the risk of colorectal cancer [54]. These three drugs have shown promising therapeutic potential in cancers other than colorectal cancer. Further basic research or clinical trials could be conducted to investigate the therapeutic effects of these three drugs in colorectal cancer. In contrast, sorafenib, cisplatin, fluorouracil, erlotinib, dasatinib, doxorubicin, etoposide, etoposide phosphate, hydroquinone, irinotecan, resveratrol, vinorelbine, oxaliplatin, and gemcitabine have been used for colon cancer treatment, but not for psoriasis treatment. Some of these drugs can potentially be effective in treating psoriasis. The primary mechanisms of these antitumor drugs are inhibiting tumor angiogenesis and suppressing tumor cell proliferation. Theoretically, these mechanisms could also be applicable to inhibiting angiogenesis and epidermal cell proliferation in psoriasis. However, these drugs often have significant side effects, such as gastrointestinal reactions, nephrotoxicity, and bone marrow suppression, which limit their use in psoriasis. There is limited research on the use of these drugs for psoriasis, with only a few case reports available. For example, there are case reports suggesting that stealth liposomal doxorubicin and carboplatin can treat psoriatic lesions in ovarian cancer patients [55]. Among these, resveratrol (3,5,4′-trihydroxy-trans-stilbene) has been relatively well-studied in the context of psoriasis. In addition to its anticancer, antiaging, and antimicrobial properties, resveratrol has anti-inflammatory and antioxidant effects that can influence the molecular pathways of inflammatory skin diseases [56]. Studies in animal and cell models have shown that resveratrol and its derivatives can effectively treat psoriasis, although the exact mechanisms are not yet clear. Moreover, resveratrol has few side effects, with high doses potentially causing gastrointestinal reactions. Further clinical trials are needed to verify its therapeutic effects in psoriasis. Further clinical experiments are necessary to verify these predictions.

## 5. Conclusions

In summary, our study primarily used bioinformatic methods to explore the shared genetic mechanisms between psoriasis and colon cancer and to identify the hub genes and pathways between these two diseases. We predicted the hub TFs and studied the expression of these hub genes and hub TFs in other cancers. It is shown that there are many coacting genetic mechanisms between psoriasis and cancer. Furthermore, we analyzed the diagnostic value and immune infiltration of hub genes and predicted therapeutic drugs. However, these hub genes, hub TFs, and predicted drugs require further experimental validation, which will be the focus of our future work.

## Figures and Tables

**Table 1:** Genes ranked first by the MCC algorithm using the cytoHubba plugin in the Cytoscape software.

**Differentially expressed genes obtained using the limma R package method**	**Shared genes obtained using the WGCNA method**
AURKA DLGAP5 NCAPG CCNB1 NDC80 BUB1B TTK CCNB2 CCNA2 KIF2C AURKB TPX2 TOP2A ASPM BUB1 KIF20A	NDC80 BUB1B RRM2 TTK MAD2L1 CCNB2 CDCA8 AURKB TOP2A CDK1 ASPM BUB1 KIF20A AURKA DLGAP5 NCAPG CCNB1 KIF11

**Table 2:** Six predicted TFs identified from the TRRUST website.

**Key TF**	**Description**	**# of overlapped genes**	**p** **value**	**Q** **value**	**List of overlapped genes**
E2F4	E2F transcription factor 4, p107/p130-binding	3	4.49e-07	2.69e-06	TTK, CCNB1, AURKB
E2F1	E2F transcription factor 1	4	1.65e-06	4.96e-06	CCNB1, AURKB, AURKA, TOP2A
E2F3	E2F transcription factor 3	2	3.40e-05	6.81e-05	CCNB1, AURKA
YBX1	Y box binding protein 1	2	0.000189	0.000283	TOP2A, CCNB1
BRCA1	Breast cancer 1, early onset	2	0.000685	0.000821	ASPM, CCNB1
TP53	Tumor protein p53	2	0.0055	0.0055	CCNB2, CCNB1

## Data Availability

All data generated or analyzed during this study are included in this published article.
